# The Leuven late life depression (L3D) study: PET-MRI biomarkers of pathological brain ageing in late-life depression: study protocol

**DOI:** 10.1186/s12888-021-03063-y

**Published:** 2021-01-28

**Authors:** Louise Emsell, Maarten Laroy, Margot Van Cauwenberge, Thomas Vande Casteele, Kristof Vansteelandt, Koen Van Laere, Stefan Sunaert, Jan Van den Stock, Filip Bouckaert, Mathieu Vandenbulcke

**Affiliations:** 1 Geriatric Psychiatry, University Psychiatric Center KU Leuven, B-3000 Leuven, Belgium; 2 KU Leuven, Leuven Brain Institute, Department of Neurosciences, Neuropsychiatry, B-3000 Leuven, Belgium; 3 KU Leuven, Department of Imaging & Pathology, Translational MRI, B-3000 Leuven, Belgium; 4 Department of Neurology, University Hospitals Leuven, Herestraat 49, B-3000 Leuven, Belgium; 5 Academisch Centrum voor ECT en Neuromodulatie (AcCENT), University Psychiatric Center KU Leuven, Kortenberg, Belgium; 6 Department of Nuclear Medicine and Molecular Imaging, University Hospitals Leuven, Herestraat 49, B-3000 Leuven, Belgium; 7 Department of Radiology, University Hospitals Leuven, Herestraat 49, B-3000 Leuven, Belgium

**Keywords:** Late life depression, Ageing, Neurodegeneration, Synaptic density, Tau, Amyloid, PET, MRI, Brain, ECT

## Abstract

**Background:**

Major depressive disorders rank in the top ten causes of ill health in all but four countries worldwide and are the leading cause of years lived with disability in Europe (WHO). Recent research suggests that neurodegenerative pathology may contribute to the development of late-life depression (LLD) in a sub-group of patients and represent a target for prevention and early diagnosis. In parallel, electroconvulsive therapy (ECT), which is the most effective treatment for severe LLD, has been associated with significant brain structural changes. In both LLD and ECT hippocampal volume change plays a central role; however, the neurobiological mechanism underlying it and its relevance for clinical outcomes remain unresolved.

**Methods:**

This is a monocentric, clinical cohort study with a cross-sectional arm evaluating PET-MR imaging and behavioural measures in 64 patients with LLD compared to 64 healthy controls, and a longitudinal arm evaluating the same imaging and behavioural measures after 10 ECT sessions in 20 patients receiving ECT as part of their normal clinical management. Triple tracer PET-MRI data will be used to measure: hippocampal volume (high resolution MRI), synaptic density using [^11^C]UCB-J, which targets the Synaptic Vesicle Glycoprotein 2A receptor, tau pathology using [^18^F]MK-6240, and cerebral amyloid using [^18^F]-Flutemetamol, which targets beta-amyloid neuritic plaques in the brain. Additional MRI measures and ultrasound will assess cerebral vascular structure and brain connectivity. Formal clinical and neuropsychological assessments will be conducted alongside experience sampling and physiological monitoring to assess mood, stress, cognition and psychomotor function.

**Discussion:**

The main aim of the study is to identify the origin and consequences of hippocampal volume differences in LLD by investigating how biomarkers of pathological ageing contribute to medial temporal lobe pathology. Studying how synaptic density, tau, amyloid and vascular pathology relate to neuropsychological, psychomotor function, stress and ECT, will increase our pathophysiological understanding of the in vivo molecular, structural and functional alterations occurring in depression and what effect this has on clinical outcome. It may also lead to improvements in the differential diagnosis of depression and dementia yielding earlier, more optimal, cost-effective clinical management. Finally, it will improve our understanding of the neurobiological mechanism of ECT.

**Trial registration:**

ClinicalTrials.gov Identifier: NCT03849417, 21/2/2019.

**Supplementary Information:**

The online version contains supplementary material available at 10.1186/s12888-021-03063-y.

## Background

Gaining insight into the pathogenesis of Late Life Depression (LLD) is crucial for treatment and prevention. Besides genetic vulnerability, environmental factors, coping strategies, and medical comorbidities, brain ageing and its effect on neural circuits involved in emotion regulation and cognitive functioning contributes to the development of depressive symptoms later in life [1]. Ageing is associated with multiple deleterious and dynamic processes in the brain which occur in parallel at different scales. These include a global loss of neurons and synapses manifesting as grey matter atrophy, deterioration in white matter leading to impaired neural connectivity, increased vascular pathology which impairs brain metabolism and promotes inflammatory processes, and the accumulation of toxic proteins such as beta-amyloid and hyperphosphorylated-tau. The idea that pathological brain ageing constitutes an important pathway to LLD is largely based on evidence that LLD is associated with an increased risk of developing cognitive decline and dementia [2]. Complementary models exist to explain the relationship. The increased risk in early-onset depression has mainly been related to stress, whereby the physiological effects of stress accelerate brain ageing (e.g. grey matter loss) and increase vulnerability to cognitive decline [3]. Cognitive decline in late-onset depression is often seen as support for the “neuropsychiatric model” whereby depression is a prodromal symptom of a neurocognitive disorder. Although the neuropsychiatric and stress models propose distinct theories, there is increasing evidence that stress interacts with neurodegeneration and cerebrovascular disease, enhancing pathological brain ageing [4]. According to the original neuropsychiatric model, lower hippocampal volume in LLD is mainly caused by preclinical or prodromal Alzheimer’s disease given that in a significant proportion of patients, LLD precedes the onset of dementia, particularly Alzheimer’s disease (AD) [5]. Moreover, higher amyloid beta burden is associated with increasing anxious-depressive symptoms over time in cognitively normal older individuals [6]. However, recent evidence from amyloid positron emission tomography studies have challenged the idea that amyloid pathology is central to the neurobiology of LLD [7, 8].

Suspected non-amyloid pathology (SNAP) is a biomarker-based concept that applies to individuals with normal levels of amyloid-β in the brain, but in whom other biomarkers of neurodegeneration are present [9]. Tau protein is abundant in neurons and plays an important role in promoting microtubule assembly and stabilizing microtubule networks. Pathological hyperphosphorylation of tau renders it insoluble and leads to its aggregation in neuronal soma and as neurofibrillary tangles. Whilst tauopathy plays a central role in AD, medial temporal tau pathology is also considered as the most important constituent of SNAP [9]. Intriguingly, depressive symptoms have recently been related to tau accumulation [10]. Moreover, animal studies suggest that stress, an important depression risk factor, may be synaptotoxic through hyperphosphorylated tau accumulation in the synapse [11]. Following the development of the next generation tau tracer [^18^F]MK-6240 which targets tau associated with neurofibrillary tangles [12] and [^11^C]UCB-J, which targets the Synaptic Vesicle Glycoprotein 2A receptor to estimate synaptic density [13], it is now possible to investigate the relationships between SNAP, primary age related tauopathy (PART) [14] and stress in LLD.

One therapeutic intervention that has a profound effect on medial temporal lobe structure in LLD is electroconvulsive therapy (ECT). ECT is a safe, rapid-acting antidepressant therapy that has repeatedly been shown to induce a transient increase in hippocampal volume [15, 16]. However, the exact origin of these structural changes and their implications for the dramatic clinical improvement (and transient cognitive side-effects) associated with ECT remains unknown. Based on preclinical research, the leading hypothesis is that volumetric changes reflect changes in synaptic plasticity [17]. For example, repeated electroconvulsive seizures increase the total number of synapses in adult male rat hippocampus [18] and increased expression of the synaptic plasticity proteins neural cell adhesion molecule 1 (NCAM1) and the presynaptic protein synapsin 1 (SYN1) have been reported in hippocampus and prefrontal cortex [19]. The translation of these preclinical findings to patients being treated with ECT is critical for understanding its mechanism of action, yet prior to the availability of [^11^C]UCB-J it was not possible to measure synaptic density in humans in vivo.

## Methods: Design

### Study aims and objectives

This study aims to shed light on how non-amyloid pathology may contribute to LLD pathology, identify the origin of LLD associated medial temporal lobe atrophy, and relate PET-MR imaging biomarkers to therapeutic response to ECT. Additionally, it aims to investigate the link between stress and MTL pathology. In order to achieve these aims, a number of objectives and associated hypotheses are proposed.

### Primary objectives

#### Objective 1

To examine whether lower hippocampal volume in LLD is associated with decreased synaptic density. *Hypothesis: Hippocampal volume will be positively correlated with synaptic density* i.e. *a lower hippocampal volume will be associated with a lower accumulation of MTL [*^*11*^*C]UCB-J.*

#### Objective 2

To study the relationship between medial temporal synaptic density and tau accumulation in LLD. *Hypothesis: Hippocampal volume will be negatively correlated with tau accumulation* i.e. *a lower hippocampal volume will be associated with a higher accumulation of MTL [*^*18*^*F]MK-6240.*

#### Objective 3

To examine whether hippocampal volume increase following ECT is associated with increased synaptic density. *Hypothesis: Hippocampal volume increase following ECT will be positively correlated with synaptic density* i.e. *Higher hippocampal volume will be associated with higher binding of MTL [*^*11*^*C]UCB-J following ECT.*

### Secondary objectives

#### Objective 4

(a) To study the relationship between medial temporal tau accumulation and the presence of white matter pathology (including WML) in tracts that are connected with the hippocampus. *Hypothesis: White matter pathology, such as WML and microstructural deterioration in MTL associated fibre tracts will be positively correlated with tau accumulation.*

#### Objective 5

To investigate whether cardiovascular risk factors are associated with biomarkers of pathological brain ageing e.g. increased white matter lesions, atrophy, and tau accumulation, and can be used in multivariate models to predict clinical outcomes. *Hypothesis: pathological ageing and microvascular damage are associated with cardiovascular risk factors and predict worse clinical outcomes in LLD.*

#### Objective 6

To relate medial temporal pathology to stress by means of ecological momentary assessment (EMA) combined with wearable biometric data collection devices and fMRI based functional connectivity measures. Hypothesis: Lower hippocampal volume and disrupted MTL functional connectivity will be associated with higher reactivity to stress and negative affect measures derived from EMA and physiological monitoring.

### Study design

#### Study setting

The study will take place in an academic hospital in Belgium and will involve data collection at two sites: the University Hospitals Leuven and the Department of Geriatric Psychiatry at the University Psychiatric Center KU Leuven. The main sponsor of the study is the KU Leuven in Belgium.

## Methods: participants, interventions, and outcomes

### Participants

A consecutive series of 64 patients (see power analysis) with late-life depression will be recruited from the Department of Geriatric Psychiatry of the University Psychiatric Center KU Leuven, Belgium. We will also include 64 age- and gender-matched healthy comparison subjects who have no prior or current depressive illness or cognitive impairment, as determined by formal clinical (psychiatric and neurological) and neuropsychological assessment (details below). Exclusion criteria will the same as for the patient group. All participants will be genotyped to determine apolipoprotein ε (APOE) status and need to provide written informed consent prior to enrolment in the study in accordance with the Declaration of Helsinki. Selection criteria are outlined below.

*Inclusion criteria*

- Age over 60 years old
- Diagnosis of late-life depression according to DSM 5 (LLD group)
- No evidence of major neurological disorder

*Exclusion criteria*

- A history of any major disease that may interfere with the investigations (especially liver and kidney disease, or uncontrolled diabetes) or cancer;
- Any history of a major neurological disorder, in particular Alzheimer’s disease, Parkinson’s disease, stroke or TBI;
- A history or evidence of psychiatric disease, as assessed by clinical interview (control group).
- A current user (including “recreational use”) of any illicit drugs, including cannabis, or a history of drug or alcohol abuse;
- Chronic use of medication that has central nervous system effects (e.g. strong painkillers such as opioids)
- Exposure to ionizing radiation (> 1 mSv) in other research studies within the last 12 months;
- Contra-indication for MRI scanning;
- Claustrophobia or intolerance to confinement during PET-MRI scanning procedures; not being able to lie still for 60 min inside the scanner;
- Inability to understand the study procedures;
- Unwillingness or inability to perform all of the study procedures, or being considered unsuitable in any way by the principal investigator;
- ECT within the last 3 months before enrolment

### Interventions

In a subgroup of patients selected according to the clinical need for ECT (estimated *n* = 20, based on experience with earlier studies, see power analysis), ECT will be administered twice a week with a constant-current brief-pulse device (Thymatron System IV, Somatics, IL, USA). Anaesthesia will be achieved with intravenous administration of etomidate (0.2 mg/kg) and succinylcholine (1 mg/kg). Motor and EEG seizure will be monitored to ensure adequate duration and quality. Subjects will all be treated with right unilateral (RUL) ECT with stimulus intensity 6 times the initial seizure threshold (ST), as determined by empirical dose titration at the first treatment, until remission (Montgomery-Åsberg Depression Rating Scale (MADRS) < 10 in two consecutive ratings with a week interval). Subjects who failed to respond to RUL ECT after the sixth treatment will be switched to bitemporal ECT (1.5xST) (Dutch Guidelines for ECT [20]). The clinical criteria/indications for ECT are determined by the treating psychiatrist and include acute suicidality with high risk of acting out suicidal thoughts, psychotic features, rapidly deteriorating physical status due to complications from the depression (e.g. poor oral intake), history of poor response to medications, history of good response to ECT, patient preference, when the risks of standard antidepressant treatment outweigh the risks of ECT, particularly in medically frail or elderly patients, and catatonia.

### Outcomes

The primary outcome measures for the cross-sectional part of the study are **hippocampal volume**, the amount of cerebral hyperphosphorylated **tau** in neurofibrillary tangles, and **synaptic density** (SV2A glycoprotein) as determined by SUVR values derived from uptake of the PET tracers 18F-MK-6420 and 11C-UCB-J respectively. The primary outcome measures for the longitudinal part of the study are **change in hippocampal volume, tau and synaptic density following ECT** as determined by a baseline measurement prior to ECT and a second measurement after 10 ECT sessions. A fixed number of sessions was chosen to reduce bias due to differences in treatment duration whilst ensuring adequate clinical response within the study population. The relationship between the primary and other outcome measures and the study objectives are outlined in Table 1. This study will additionally generate numerous outcome variables that can be used to address secondary hypotheses relating, but not limited to, the objectives in this study.

**Table 1:** Research questions and outcome data to be used to address the study objectives

Objective	Research Question	Experimental data	Variables
1	Hippocampal atrophy associated with SD?	**MRI: 3D T1** BRAVO & **3D T2** (morphometry & PET pre-processing) **PET:** ^**11**^**C-UCB-J PET** (SV2a: SD)	Hippocampal volume ^11^C-UCB-J SUVR
2	Tau associated with SD in hippocampus?	**MRI: 3D T1 BRAVO** & **3D T2** (as above) **PET:** ^**18**^**F-MK-6240** (tau)	Hippocampal volume ^18^F-MK-6240 SUVR
3	ECT-induced ΔGM in hippocampus associated with ΔSD?	**MRI: 3D T1 & 3D T2** (as above + post ECT) **PET:** ^**11**^**C-UCB-J PET** (SD)	Δ Hippocampal volume Δ^11^C-UCB-J
4	Tau associated with MTL WM damage?	**MRI: 3D FLAIR** (to define + quantify WML) **SWI & ASL** (perfusion/vascular pathology) **multi-shell diffusion** (DKI, NODDI + tractography) **PET:** ^**18**^**F-MK-6240** (tau)	Total + regional cerebral WML volume ^18^F-MK-6240 SUVR WM microstructural damage (e.g. MK, FA, ND), MTL WM fibre bundles
5	Pathological brain ageing mediated by CV risk factors?	**MRI: 3D T1 & T2** (as above) **MRI: 3D FLAIR** (to define + quantify WML) **SWI & ASL** (perfusion/vascular pathology) **PET:** ^**11**^**C-UCB-J PET** (SD) **PET:** ^**18**^**F-MK-6240** (tau) **Doppler ultrasound** (carotid artery) **Blood biomarkers** (lipids, glucose)	GM volume/cortical thickness Total + regional cerebral WML volume ^11^C-UCB-J & ^18^F-MK-6240 SUVR Intima-media thickness Serum lipids (HDL, LDL), glucose (HbA1c) Motor function metrics
6	Stress ➔ ↑tau & ↓SD ➔ MTL functional connectivity?	**MRI: resting-state fMRI** (fc connectivity) **3D T1** *(as above)* **PET:** ^**11**^**C-UCB-J PET** (SD) **PET:** ^**18**^**F-MK-6240** (tau) **Stress measures/EMA**	Hippocampal +amygdala volume ^11^C-UCB-J & ^18^F-MK-6240 SUVR fc in MTL associated resting-state networks physiological stress metrics / EMA response

Abbreviations: *SD* synaptic density, *ECT* electroconvulsive therapy; *GM* Grey matter, *MRI* magnetic resonance imaging, *SUVR* Specific uptake value ratio, *MTL* Medial temporal lobe, *WM* White matter, *WML* WM lesion, *SWI* susceptibility weighted imaging, *ASL* arterial spin labelling, *PET* positron emission tomography, *MK* mean kurtosis, *FA* fractional anisotropy, *ND* neurite density, *HDL* high density lipoprotein, *LDL* low density lipoprotein, *EMA* ecological momentary assessment, *HbA1c* haemoglobin A1c

### Participant timeline

**Fig. 1:**
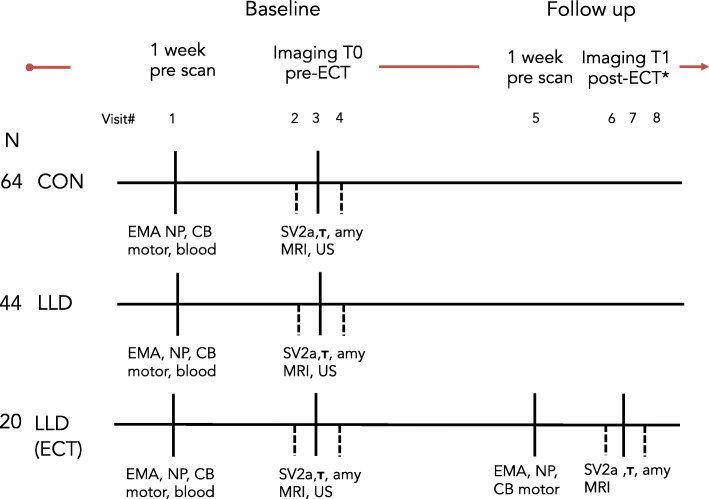
Schematic timeline for L3D data collection. Figure legend: amy: Amyloid PET, blood: blood sampling (apolipoprotein □4 genotyping, blood glucose and serum lipids), CB: Chill Band wristband monitoring, CON: control subject, ECT: electroconvulsive therapy, EMA: ecological momentary assessment, LLD: late-life depression, MRI: magnetic resonance imaging, motor: neurological and task based assessment of motor function, NP: neuropsychological testing and psychiatric intake, SV2A: synaptic density PET with synaptic vesicle 2A tracer, t:tau PET, US: doppler ultrasound (intima media thickness) *following the 10th ECT treatment

Data will be collected from participants during four visits for the cross-sectional part of the study, and eight visits for the longitudinal part (ECT group). The first visit will comprise the intake, obtaining informed consent, clinical neurological, psychiatric and neuropsychological assessments, and venous blood sampling for genotyping, glycated hemoglobin (HbA1c) and serum lipid measurements. At the end of the first visit, the participants also receive the Chill Band (a wristband monitoring device) and a dedicated smartphone for ecological momentary assessment (EMA). PET-MR imaging will be conducted at the second, third and fourth visit. Cerebrovascular ultrasound will also be performed during one of these visits (Fig. 1).

### Sample size

Our proposed sample size of 128 participants is based on the following calculations. As our hypotheses assume baseline group differences between controls and patients in hippocampal volume and synaptic density, and increases in hippocampal volume following ECT, we have based our power calculations on t-tests to determine such differences, rather than on correlations.

(1) Based on the results of our previous study [7] (Group 1 mean = 6396.251 mm^3^, Group 2 Mean = 6724.461 mm^3^, Group 1 Standard Deviation = 711.49 mm^3^ Group 2 Standard Deviation = 763.866 mm^3^), using the STATA statistical package POWER procedure, to have a power of at least 0.80 (0.804) to detect a group mean difference in normalized total hippocampal volume using a two-sample t-test with α-level of 0.05 (one-tailed), assuming a balanced design with equal group sizes, requires 64 subjects per group, i.e. 128 subjects in total.

(2) To examine hippocampal volume changes after ECT treatment we will assess normalized total hippocampal volume on a random sample of patients before and after the last ECT treatment. Based on the expected mean difference in normalized total hippocampal volume (− 188.333 mm^3^), pre 792.5462 mm^3^ and post standard deviations 864.075 mm^3^, and the correlation between pre and post volume hippocampal measurements (0.92908) from our previous study [15], to achieve 80% power (0.814) at an α-level of 0.05 (one-tailed) requires 20 subjects.

(3) Based on the results of a recent study [21] investigating differences in ^11^C-UCB-J binding in older healthy controls and AD patients with mild cognitive impairment and mild dementia (mean age = 73 years), (Group 1 mean = 0.87, Group 2 Mean = 1.47, Group 1 Standard Deviation = 0.5 Group 2 Standard Deviation = 0.37), to have a power of at least 0.80 (0.826) to detect a group mean difference in ^11^C-UCB-J (synaptic density) using a two-sample t-test with α-level of 0.05 (one-tailed), assuming a balanced design with equal group sizes, requires 8 subjects per group, i.e. 16 subjects in total.

### Recruitment

Healthy, control participants will be recruited from the local community by distributing posters, flyers and information brochures to local stores as well as recreational, cultural and political clubs for the elderly, and also via local newspaper and online advertising. Suitable participants with LLD will be recruited by referral from their treating psychiatrist within the university psychiatric hospital network.

## Methods: data collection and statistical analysis

### Imaging

All data will be collected on site at either the University Psychiatric Center KU Leuven or Department of Nuclear Medicine at the University Hospital Leuven. Imaging data will be acquired on GE Signa 3 T time-of-flight PET-MR (^11^C-UCB-J*,^18^F-MK-6240*) and Siemens Biograph True Point PET-CT (^18^F-flutemetamol*) systems. A venous catheter line will be placed in one arm for IV tracer injection. For [^18^F]MK-6240, static PET imaging will be acquired for 30 min, starting 90 min after tracer injection. For [^11^C] UCB-J PET, static image acquisition will be initiated at 60 min after tracer injection for 30 min. For [^18^F] flutemetamol, static PET data acquisition will be started at 90 min after tracer injection and the data will be acquired for 30 min. The PET data will be reconstructed using iterative reconstruction. Attenuation correction will be done using a zero-echo-time (ZTE) MR scan. ZTE allows MR imaging of bone, which enables better skull segmentation, tissue assignment and accurate PET quantification. All reconstructed PET data will incorporate partial volume effect correction based on the simultaneously acquired 3D T1-weighted MR images.

Simultaneous MR data acquisition will be spread over two PET-MRI sessions to minimize participant burden and will include whole brain structural 3D T1 (BRAVO) and 3D T2 images (both 1 mm × 1 mm × 1 mm) for morphometry and PET pre-processing; a 3D T2 FLAIR for e.g. quantification of WM lesions (1 mm × 1 mm × 1 mm) and high-resolution T2 imaging of the hippocampus (0.5 mm × 0.5 mm × 2 mm). Multi-shell DWI will be acquired to calculate quantitative measures of WM microstructure and will be analysed using the MRTrix3 and NODDI toolboxes. Perfusion and vascular pathology will be assessed using arterial spin labelling (ASL) and susceptibility weighted imaging (SWI). Age related WM changes and signs of small vessel disease will be assessed using FLAIR, DWI, T1 and SWI scans, and reported in accordance with the “standards for reporting vascular changes on neuroimaging” (STRIVE) guidelines [22]. The main MRI derived outcome measure, hippocampal volume, will be calculated using an automated segmentation algorithm applied to T1 and T2 weighted images e.g. using FreeSurfer v7.1. The SPM CAT12 toolbox will be used to assess voxel-based group differences and associations between grey matter volume and cortical thickness, synaptic density and tau accumulation. Resting state fMRI data will be pre-processed with fMRIPrep 20.2 LTS and analysed using the *conn* toolbox and will focus on alterations in functional networks associated with the medial temporal lobe.

Cerebrovascular ultrasound of the common carotid artery and internal carotid artery will be performed in all participants using a colour-coded phased-array ultrasound system (EPIQ 5, Phillips). Intima-media thickness will be measured in B mode automatically on longitudinal plane images measured 1 cm below the carotid bulb. Transcranial ultrasound (TCS) will be performed to obtain substantia nigra intensity area measurement, third ventricle and MTL measurements and nucleus raphe echogenicity rating [23, 24]. The vascular ultrasound and intracranial ultrasound will take place consecutively in one session that has an estimated total time of 15 min.

### Clinical, neuropsychological and psychomotor assessment

All subjects will undergo a general medical, psychiatric and neurological assessment. Presence of psychiatric disease and symptoms will be determined according to DSM 5 criteria and Neuropsychiatric Inventory (NPI) respectively. Depression severity will be assessed using the Montgomery-Åsberg Depression Rating Scale (MADRS) and Geriatric Depression Scale (GDS). Apathy will be assessed with the Apathy Evaluation Scale (AES). The following neuropsychological examinations to test memory, language and executive functioning as performed in our previous studies (e.g. De Winter et al), including the Mini-Mental State Examination (MMSE), Rey Auditory Verbal Learning Test (RAVLT), Raven’s Coloured Progressive Matrices (RCPM), Trail-making test (TMT), digit span, animal verbal fluency (AVF) and Boston Naming Test (BNT). Cardiovascular disease risk will be assessed using the Framingham Heart Study general cardiovascular disease (FHS-CVD) risk score and will include clinical assessment of blood pressure (after 5 min supine as well as after 1 and 3 min standing), serum lipids and glycated haemoglobin (HbA1c). In the ECT group, autobiographical memory will be tested pre and post ECT using the Autobiographic Memory Interview.

Motor performance will be investigated clinically using the CORE rating scale as well as the unified Parkinson’s disease rating scale motor part III (UPDRS III). The CORE (referring to the “core” features of melancholia) is used to assess psychomotor symptoms in a psychiatric population and comprises 18 observable features which are rated on a four-point scale (0–3) in order to dimensionally quantify the severity of psychomotor symptoms in depression [25]. The UPDRS III is an observer rated 14 item five-point rating scale (0–4) that assesses fine and gross motor function as well as postural stability and gait in a neurological population. Frontal release signs will be tested by an experienced clinician as a part of a standardized neurological examination. Coordination and balance will be investigated using the scale for the assessment and rating of ataxia (SARA). The clinical assessment of motor performance as well as coordination and balance will be filmed to perform a post-hoc second rating by an investigator blinded to the participant’s group membership.

To experimentally assess fine motor function, a line-drawing task in a cued and uncued setting will be performed on a tablet. This drawing and writing task will take place on a 2- in − 1 graphical tablet (WACOM mobile studio pro 13) using MovalyzeR software (NeuroScript LLC, Tempe, AZ, USA), with simultaneous eye tracking using the tobii TX 300 infrared eye tracker (Tobii AB, Danderyd, Stockholm, Sweden). The task will be performed in a comfortable environment and has an estimated duration of 20 min, preceded by a 10 min ‘try-out’ session to become familiar with the digital pen. Fine motor performance will also be tested in a non-digital environment with the unmodified Purdue Pegboard task. Experimental measurement of gross motor activity in daily life will be measured using an actimeter within the Chill Band. The Chill Band allows tracking and storage of activity by a built-in 3-axis accelerometer and gyroscope which will be used to quantify the quantity and quality (amplitude, speed) of gross movement.

Lifetime stress will be assessed using the self-report childhood trauma questionnaire (CTQ) - short form and the psychiatric epidemiology research interview (PERI). Current stress will be assessed using electronic ecological momentary assessment (EMA), which provides good compliance in older populations [26] and has been successfully performed in late life and major depression [27]. During six consecutive days prior to their first scan, participants will be prompted by a smartphone alert to fill out a brief digital questionnaire on the smartphone assessing their current mood, stressful events, interoceptive awareness, social context and their appraisal of the context, at a frequency of 8 times a day at a random timepoint in each of 8 90-min time intervals between 8:00 and 22:00.

In accordance with previous research Negative affect (NA) will be calculated using a weighted mean score of ESM items “I feel insecure”, “I feel anxious”, “I feel down”, “I feel guilty” and “I feel lonely”, each rated on a 7-point Likert scale (1 = not at all; 7 = very). For the stress assessment, participants will be asked to think about the most important event that happened since the previous alert and then report “How pleasant was this event?” (− 3: very unpleasant; 3: very pleasant). If the event is rated lower than 0 (i.e. unpleasant events) the event will be considered stressful. Reactivity to stress will be calculated as the intra-individual effect size of event unpleasantness on NA [27, 28]. In addition to the stress assessment, participants are required to indicate the momentary social context and their appreciation thereof. The questionnaire ends with a question on interoceptive awareness. These EMA measures are valid regarding subjective stress response and correlate with other behavioural and physiological stress measures [29]. To link the data from the EMA of event-related behaviour to physiological stress measures, we will collect peripheral physiological data by means of the Chill Band developed by IMEC which allows tracking and storage of skin conductance and skin temperature, in addition to activity. Dedicated algorithms (to enhance signal quality and to extract stress related features) are available and will be further optimized for the purpose of this study.

### Blood sampling

Venous blood samples will be collected for serum, plasma, DNA and RNA extraction. After collection, blood tubes will be stored for 30 min at room temperature, in a horizontal (serum tubes) or vertical position (all other tubes). Plasma and serum tubes will be centrifuged at 4 °C for 10 min. Plasma and serum will be carefully removed using a pipette with 800 μl per aliquot. All samples will be stored in a coded manner at − 80 °C in the UZ/KU Leuven biobank for further research use in the current project.

### Statistical analysis

The following section describes the specific analysis of imaging data as this will be used to derive the primary outcome measures hippocampal volume, synaptic density and cerebral tau burden. Note that in addition to these analyses, conventional statistical tests (e.g. t-tests, correlations, regression, and their longitudinal equivalents, paired-T, linear mixed models with repeated measures) will be applied to address the research questions outlined in objectives 1–6 and in Table 1 in accordance with how the data align with statistical assumptions (e.g. normality, homogeneity etc.).

In this prospective longitudinal study, we will first analyse individual modalities and sequences independently in univariate analyses to address the different modality and underlying physiological/morphological research questions. More specifically, to investigate whether hippocampal volume is associated with tau and synaptic density we will perform voxel-wise linear regression analysis in SPM12, including age and total intracranial volume as covariates. To investigate whether hippocampal volume change following ECT is associated with a corresponding change in synaptic density we will apply a longitudinal linear mixed model controlling for age, in which hippocampal volume and hippocampal synaptic density are dependent variables.

Multivariate imaging analysis methods based on machine learning and deep learning will be applied subsequently to investigate relationships between the different modalities and their predictive value for specific symptom dimensions, diagnostic classification and clinical outcomes [30]. Multiple comparisons correction will be applied and will depend on the type of analysis. Missing data will be described and accounted for using a model appropriate for the purpose. For example, using the direct likelihood method and/or multiple imputation [31, 32].

Validated image analysis software incorporated into in-house MATLAB and Python-based processing pipelines will be used to both pre-process and analyse the data, including, but not limited to PMOD, SPM12/CAT12, Freesurfer, PetSurfer, and KUL neuroimaging tools. Where possible “brain imaging data structure” (BIDS) compatibility will be ensured to facilitate quality assurance, reproducibility and data-sharing. Both whole-brain voxel-wise analysis and atlas-based region of interest analysis will be used to relate hippocampal volume to cerebral tau and synaptic density distribution, and to explore associations between these variables and measures of mood (e.g. based on, but not limited to GDS) and cognition (e.g. based on, but not limited to MMSE, RAVLT).

## Discussion

As the interplay between biological, social, psychological and behavioural factors is central to the aetiology of depression, we will employ an integrated biological and psychosocial approach in this study. Alongside our imaging data, we will collect clinical, neuropsychological and behavioural data combined with biometric data from a wearable collection device to monitor stress. This will allow us to situate the relationship between imaging markers (e.g. tau pathology) and depression phenotype in a more authentic clinical context. However, the sheer amount and variety of data being collected brings many challenges with it. Firstly, it requires onsite access to state-of-the-art clinical, nuclear medicine, MR imaging, engineering and biostatistics facilities as well as extensive interdepartmental collaboration and coordination between a wide range of personnel in these different domains. For example, data collection takes place on at least four occasions, and in the longitudinal study, eight occasions in a period of 2 months, and involves coordination not only between several clinicians and research staff, but also the administrative and technical staff involved in tracer production and transport, PET-MR imaging data collection, as well as processing and storage of blood samples. Secondly, but of no lesser importance are the challenges associated with recruiting and retaining participants who are currently experiencing a depressive episode, particularly those who are severely depressed and awaiting ECT. In this context our team has a great deal of experience supporting such individuals and facilitating their participation in the most comfortable conditions possible. For example, by involving carers, and adapting the study protocol to accommodate bedside data collection when possible. Nevertheless, given the demands of the study and as with all clinical studies, it is probable that some participants will not have a complete dataset. Thirdly, for practical reasons it is not possible to control for sample heterogeneity with regard to medication use and subclinical medical comorbidity in a geriatric population. Whilst this naturalistic approach makes the sample authentic, it means that it may not be possible to generalise the findings to all individuals in this age-range or those with LLD. Fourth, whilst we are confident that the proposed sample size based on our power calculation will be sufficient to answer the main research questions described in the methods section, we cannot assume that it will also be sufficient to address the many sub-analyses that may arise from a varied dataset of this size or control for confounds alluded to previously. However, by harmonizing our protocol with other groups and following international standards, as well as enabling data-sharing in our informed consent procedure, we will ensure that the data generated by L3D will be available to research consortia in order to facilitate analyses requiring larger samples. This will be particularly important in the context of identifying clinically useful biomarkers derived from machine learning approaches.

In summary, the L3D study will use new PET-MRI brain imaging biomarkers of neurodegeneration in combination with wearable technology to investigate how stress and neural markers of pathological brain aging such as tau protein abnormalities (tauopathy), temporal lobe grey matter loss, white matter and vascular pathology contribute to the pathophysiology of late-life depression. The results may lead to improved understanding of the neurobiology underlying both the effects of ECT on cognition and mood, and a new proteinopathy-based stratification of late-life depression.

## Supplementary Information

**Additional file 1.** STROBE checklist: Table detailing conformance of study/manuscript to STROBE criteria

## Data Availability

The datasets that will be used or analysed during the course of the study will be available from the corresponding author on reasonable request.

## Abbreviations

AD: Alzheimer’s Disease
AES: Apathy evaluation scale
APOε4: Apolipoprotein E4 allele
AMI: Autobiographic Memory Interview
ASL: Arterial spin labelling
AVF: Animal verbal fluency
BNT: Boston Naming Test
BIDS: Brain imaging data structure
CAT12: Computational anatomy toolbox
CT: Computed tomography
CTQ: Childhood trauma questionnaire
DSM-V: Diagnostic and statistical manual version 5
ECT: Electroconvulsive therapy
EMA: Ecological momentary assessment
FHS-CVD: Framingham Heart Study general cardiovascular disease risk score
FA: Fractional anisotropy
GDS: Geriatric depression scale
GM: Grey matter
HbA1C: Haemoglobin A1c
HDL: High density lipoprotein
KUL: KU Leuven
L3D: Leuven Late Life Depression (study)
LDL: Low density lipoprotein
LLD: Late life depression
MADRS: Montgomery Asberg Depression Scale
MK: Mean kurtosis
MMSE: Mini mental state examination
MRI: Magnetic resonance imaging
MTL: Medial temporal lobe
ND: Neurite density
NP: Neuropsychology.
PART: Primary age related tauopathy
PERI: Psychiatric epidemiology research interview
PMOD, PET: Positron emission tomography
RAVLT: Rey auditory verbal learning test
RCPM: Raven Coloured Progressive Matrices
SARA: Scale for the Assessment and Rating of Ataxia
SNAP: Suspected non-amyloid pathology
SPIRIT: Standard Protocol Items: Recommendations for Interventional Trials
SD: Synaptic density
STRIVE: Standards for reporting vascular changes on neuroimaging
SUVR: Specific uptake value ratio
SV2A: Synaptic vesicle 2A
SWI: Susceptibility weighted imaging
TMT: Trail-making test
UPDRS: Unified Parkinson’s Disease Rating Scale
US: Ultrasound
VBM: Voxel-based morphometry
White matter: WM
WMH: White matter hyperintensity
WML: White Matter lesion
ZTE: Zero-echo time
