# Supplementary material for: The Leuven late life depression (L3D) study: PET-MRI biomarkers of pathological brain ageing in late-life depression: study protocol
Source: BMC Psychiatry. 2021 Jan 28;21:64. doi: 10.1186/s12888-021-03063-y (PMC7845114; doi:10.1186/s12888-021-03063-y)
Supplement: Supplementary file 1 — Additional file 1. STROBE checklist: Table detailing conformance of study/manuscript to STROBE criteria [file 12888_2021_3063_MOESM1_ESM.docx]

**Additional File 1: STROBE checklist**

|  | Item No | Recommendation | Article location |
| --- | --- | --- | --- |
| Title and abstract | 1 | (*a*) Indicate the study’s design with a commonly used term in the title or the abstract | Page 1 |
|  |  | (*b*) Provide in the abstract an informative and balanced summary of what was done and what was found | Page 2, paragraph 2: methods. No results reported as this is a study protocol |
| Introduction |  |  |  |
| Background/rationale | 2 | Explain the scientific background and rationale for the investigation being reported | Page 3, section 1 Background |
| Objectives | 3 | State specific objectives, including any prespecified hypotheses | Page 5, section 2.1 Study aims and objectives |
| Methods |  |  |  |
| Study design | 4 | Present key elements of study design early in the paper | Page 1, abstract, Page 6, section 2.2 Study design, Figure 1: Schematic timeline |
| Setting | 5 | Describe the setting, locations, and relevant dates, including periods of recruitment, exposure, follow-up, and data collection | Page 6, section 2.2.1 Study setting, Figure 1: Schematic timeline |
| Participants | 6 | (*a*) *Cohort study*—Give the eligibility criteria, and the sources and methods of selection of participants. Describe methods of follow-up  *Case-control study*—Give the eligibility criteria, and the sources and methods of case ascertainment and control selection. Give the rationale for the choice of cases and controls  *Cross-sectional study*—Give the eligibility criteria, and the sources and methods of selection of participants | Page 6-7, section 2.2.2: Methods: Participants, interventions and outcomes, paragraph 1: participants, p7 Interventions, paragraph 1. Page11: Recruitment |
|  |  | (*b*) *Cohort study*—For matched studies, give matching criteria and number of exposed and unexposed  *Case-control study*—For matched studies, give matching criteria and the number of controls per case |  |
| Variables | 7 | Clearly define all outcomes, exposures, predictors, potential confounders, and effect modifiers. Give diagnostic criteria, if applicable | Page 9 Section: Outcomes |
| Data source measurements | 8 | For each variable of interest, give sources of data and details of methods of assessment (measurement). Describe comparability of assessment methods if there is more than one group | Pages 12-16, section 2.2.3 Methods: Data collection and statistical analysis, Table 1 |
| Bias | 9 | Describe any efforts to address potential sources of bias | Page 9, section; outcomes “*A fixed number of sessions was chosen to reduce bias due to differences in treatment duration whilst ensuring adequate clinical response within the study population”* |
| Study size | 10 | Explain how the study size was arrived at | Page 11-12, section 2.2.2 Methods: Participants, interventions, and outcomes, section “Sample size”, power calculation |
| Quantitative variables | 11 | Explain how quantitative variables were handled in the analyses. If applicable, describe which groupings were chosen and why | N/A (no data analysed) |
| Statistical methods | 12 | (*a*) Describe all statistical methods, including those used to control for confounding | Pages 16-17, section 2.2.3. Methods: Data collection and statistical analysis, subsection Statistical analysis |
|  |  | (*b*) Describe any methods used to examine subgroups and interactions | N/A |
|  |  | (*c*) Explain how missing data were addressed | N/A (no data analysed). Missing data approach described on page 17, paragraph 2. |
|  |  | (*d*) *Cohort study*—If applicable, explain how loss to follow-up was addressed  *Case-control study*—If applicable, explain how matching of cases and controls was addressed  *Cross-sectional study*—If applicable, describe analytical methods taking account of sampling strategy | N/A (no data analysed). Use of covariates age and total intracranial volume (section Statistical analysis). Missing data approach described on page 17, paragraph 2. |
|  |  | (*e*) Describe any sensitivity analyses | N/A |
| Results |  |  |  |
| Participants | 13 | (a) Report numbers of individuals at each stage of study—e.g. numbers potentially eligible, examined for eligibility, confirmed eligible, included in the study, completing follow-up, and analysed | N/A: Study protocol, no results |
|  |  | (b) Give reasons for non-participation at each stage | N/A: Study protocol, no results |
|  |  | (c) Consider use of a flow diagram | N/A: Study protocol, no results |
| Descriptive data | 14 | (a) Give characteristics of study participants (e.g. demographic, clinical, social) and information on exposures and potential confounders | N/A: Study protocol, no results |
|  |  | (b) Indicate number of participants with missing data for each variable of interest | N/A: Study protocol, no results |
|  |  | (c) *Cohort study*—Summarise follow-up time (e.g. average and total amount) | N/A: Study protocol, no results |
| Outcome data | 15 | *Cohort study*—Report numbers of outcome events or summary measures over time | N/A: Study protocol, no results |
|  |  | *Case-control study—*Report numbers in each exposure category, or summary measures of exposure |  |
| Main results | 16 | (*a*) Give unadjusted estimates and, if applicable, confounder-adjusted estimates and their precision (e.g. 95% confidence interval). Make clear which confounders were adjusted for and why they were included | N/A: Study protocol, no results |
|  |  | (*b*) Report category boundaries when continuous variables were categorized | N/A: Study protocol, no results |
|  |  | (*c*) If relevant, consider translating estimates of relative risk into absolute risk for a meaningful time period | N/A: Study protocol, no results |
| Other analyses | 17 | Report other analyses done—e.g. analyses of subgroups and interactions, and sensitivity analyses | N/A: Study protocol, no results |
| Discussion |  |  |  |
| Key results | 18 | Summarise key results with reference to study objectives | N/A: Study protocol, no results |
| Limitations | 19 | Discuss limitations of the study, taking into account sources of potential bias or imprecision. Discuss both direction and magnitude of any potential bias | Pages 17-19, Section 3: Discussion |
| Interpretation | 20 | Give a cautious overall interpretation of results considering objectives, limitations, multiplicity of analyses, results from similar studies, and other relevant evidence | N/A: Study protocol, no results |
| Generalisability | 21 | Discuss the generalisability (external validity) of the study results | N/A Pages 17-19, Section 3: Discussion, refers to future generalisability |
| Other information |  |  |  |
| Funding | 22 | Give the source of funding and the role of the funders for the present study and, if applicable, for the original study on which the present article is based | Page 20, section 10: Funding. |
